# Safe, accurate, prenatal diagnosis of thanatophoric dysplasia using ultrasound and free fetal DNA

**DOI:** 10.1002/pd.4066

**Published:** 2014-08-07

**Authors:** Lyn S Chitty, Asma Khalil, Angela N Barrett, Eva Pajkrt, David R Griffin, Tim J Cole

**Affiliations:** 1 Clinical and Molecular Genetics Unit, UCL Institute of Child HealthLondon, UK; 2 Fetal Medicine Unit, University College London Hospitals NHS Foundation TrustLondon, UK; 3 North East Thames Regional Genetics Laboratory, Great Ormond Street HospitalLondon, UK; 4 Fetal Medicine Unit, Academic Medical CentreAmsterdam, The Netherlands; 5 Department of Obstetrics and Gynaecology, West Herts HospitalWatford, Hertfordshire, UK; 6 Paediatric Epidemiology and Biostatistics, UCL Institute of HealthLondon, UK

## Abstract

**Objective:**

To improve the prenatal diagnosis of thanatophoric dysplasia by defining the change in fetal size across gestation and the frequency of sonographic features, and developing non-invasive molecular genetic diagnosis based on cell-free fetal DNA (cffDNA) in maternal plasma.

**Methods:**

Fetuses with a confirmed diagnosis of thanatophoric dysplasia were ascertained, records reviewed, sonographic features and measurements determined. Charts of fetal size were then constructed using the LMS (lambda-mu-sigma) method and compared with charts used in normal pregnancies and those complicated by achondroplasia. Cases in this cohort referred to our Regional Genetics Laboratory for molecular diagnosis using cffDNA were identified and results reviewed.

**Results:**

Forty-two cases were scanned in our units. Commonly reported sonographic features were very short and sometimes bowed femora, frontal bossing, cloverleaf skull, short fingers, a small chest and polyhydramnios. Limb shortening was obvious from as early as 13 weeks' gestation, with minimal growth after 20 weeks. Analysis of cffDNA in three of these pregnancies confirmed the presence of the c.742C>CT (p.Arg248Cys) or the c.1948A>AG (p.Lys650Glu) mutation in the fibroblast growth factor receptor 3 gene.

**Conclusion:**

These data should improve the accuracy of the sonographic diagnosis of thanatophoric dysplasia and have implications for reliable and safe targeted molecular confirmation using cffDNA. © 2013 The Authors. *Prenatal Diagnosis* published by John Wiley & Sons Ltd.

## INTRODUCTION

**Figure 1:**
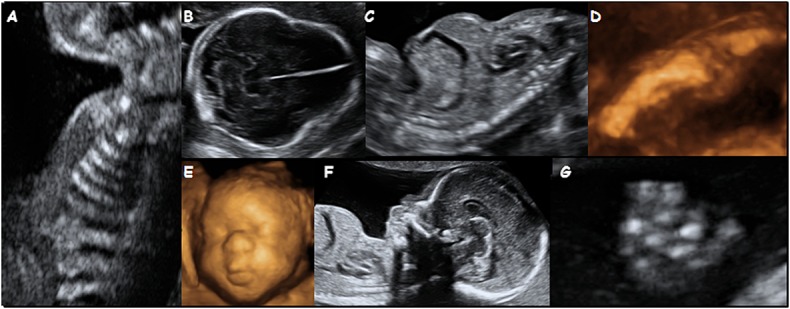
Sonographic features of thanatophoric dysplasia (TD) with a view of the thorax demonstrating short ribs (A), a view of the head in a fetus with TD II and a cloverleaf skull (B), a longitudinal view of the thorax and abdomen demonstrating the ‘champagne cork’ appearance due to the very small chest (C), a 3-D image of the femur demonstrating slight bowing (D), a 3-D view of the face (E), a sagittal view showing the frontal bossing (F) also demonstrating a very small chest and a view of a hand illustrating the short fingers (G)

Thanatophoric dysplasia (TD) is the most common lethal dominant skeletal dysplasia with an incidence of 2–3 per 100 000 births.^1^ It results from a mutation in the fibroblast growth factor receptor 3 (FGFR3) gene located on 4p16.3.^2^,^3^ There are several mutations known to cause TD, the most common being Arg248Cys, Tyr373Cys and Lys650Glu.^3^ TD may be divided into subtypes I and II, based both on molecular diagnosis and on clinical features, but there is considerable overlap between the two groups. Cases with a Lys650Glu mutation have predominantly milder radiological and histological features with straight femora and craniosynostosis and have been classified as Type II. In Type I, femora tend to be curved and craniosynostosis infrequent,^3^ resulting most commonly from the Arg248Cys or Tyr373Cys substitutions. Prenatal sonographic detection occurs traditionally in the second trimester, at the time of the routine anomaly scan when the fetus is found to have short long bones, a small chest, macrocephaly, frontal bossing, short fingers, cloverleaf skull or telephone receiver-shaped femora (Figure 1), but with increasing use of first trimester ultrasound, these features are increasingly detected earlier in pregnancy.^4^ The accuracy of prenatal diagnosis of TD based on ultrasound findings has been reported to vary from 40% to 88%.^5^–^10^

Accurate sonographic diagnosis allows for targeted molecular testing that, until recently, required analysis of fetal cells obtained following an invasive diagnostic test such as amniocentesis or chorionic villus sampling.^11^ Identification of cell-free fetal DNA (cffDNA) circulating in maternal plasma^12^ has allowed for the development of safer, non-invasive genetic prenatal diagnosis that relies on a maternal blood sample.^13^

The objectives of this study were to improve the prenatal diagnosis of TD by constructing fetal size charts for fetuses with TD, defining the frequency of other associated sonographic features in order to highlight differences with other dysplasias and to explore the possibility of developing targeted molecular analysis using cffDNA for definitive prenatal diagnosis.

## MATERIALS AND METHODS

### Development of fetal size charts and ascertainment of sonographic features

Our fetal medicine unit databases were searched from 1992 to 2011 for all fetuses that presented prenatally with short limbs where TD was the aetiology. Only those with a diagnosis confirmed either by the presence of an *FGFR3* gene mutation or by post-mortem radiology were included. Prenatal records were examined to ascertain all fetal measurements and other sonographic features. This was part of a retrospective audit of cases, and local approvals were not required.

A search of PubMed, Web of Science and EMBASE was made to identify previous citations in the English literature reporting sonographic findings in fetuses with TD. We used MeSH (medical subject heading) terms and key words related to TD and prenatal ultrasound, including ‘thanatophoric dysplasia’, ‘ultrasound’, ‘prenatal’, ‘sonography’, ‘skeletal dysplasia’, ‘limb shortening’, ‘macrocephaly’, ‘telephone-receiver femur’, ‘cloverleaf skull’, ‘lethal’ and ‘chondrodysplastic dwarfism’. Studies where data on the prenatal sonographic findings in TD could be identified were included.

### Construction of fetal size charts

Using data obtained from our own cohort of cases, we constructed charts of fetal size for head circumference (HC), abdominal circumference (AC) and long bones using the LMS (lambda-mu-sigma) method.^14^ This method models the age-changing distribution of the measurement in terms of the median and coefficient of variation after applying a suitable Box–Cox power transformation. Here, the medians were fitted as smooth curves, whereas the coefficients of variation all decreased linearly with increasing gestation. The distributions of all three measurements were skewed, requiring Box–Cox transformations of power −0.5, 3.7 and −3.9 for femur length (FL), AC and HC, respectively. The data derived were also compared with charts recommended for routine assessment of fetal size in the UK,^15^–^17^ both as centile plots and *Z*-score plots relative to these normal ranges. In addition, the femur length charts were compared with those derived for fetuses with achondroplasia.^18^

### Development of non-invasive prenatal diagnosis of TD using cffDNA

Three women in this series had maternal blood taken for analysis of cffDNA. All cases had blood taken prior to invasive procedures or termination of pregnancy. In two cases, blood was taken and plasma stored as part of an ongoing study to develop non-invasive prenatal diagnosis (NIPD) using cffDNA.^19^ In the third affected pregnancy, blood was taken prospectively and the results used to confirm the sonographic diagnosis prior to termination of pregnancy. The diagnosis was also confirmed by molecular analysis of skin cells after delivery. This woman also had blood taken in a second pregnancy, which was not affected and resulted in the birth of a normal baby. In all cases, plasma was separated from the blood cells by centrifugation at 1500 *g* for 10 min. The supernatant was then transferred to fresh tubes, ensuring that the buffy coat remained intact. The plasma was then centrifuged at 16 000 *g* for 10 min to remove any remaining cells and analysed immediately or transferred into 2 mL Lo-Bind tubes (Eppendorf, Hamburg, Germany) and stored at −80 °C until DNA extraction.

### DNA extraction

DNA was extracted from 5 mL of plasma using the QIAamp Circulating Nucleic Acid kit (Qiagen, Hilden, Germany) according to manufacturer's instructions and was eluted into a final volume of 75 μL AVE elution buffer.

### PCR from cffDNA

Primer sequences are detailed in Table 1. The primers TDIF and TDIR were used to amplify the segment of DNA that contains nucleotide c.742. Two sets of primers, namely TDIIF1 and TDIIR1, and TDIIF2 and TDIIR2, were used to amplify the segment of DNA containing nucleotide c.1948. Five microlitres of plasma DNA or 50 ng of gDNA was used as a template for each PCR, in a mix containing 1× PCR Buffer II, 3 μL MgCl_2_, 200 μM of each dNTP, 10 pmol of each primer and 0.25 μL Amplitaq Gold in a final volume of 50 μL. Cycling conditions were as follows: after an initial incubation at 95 °C for 5 min, the reaction was cycled for 10 cycles at 95 °C for 15 s, 65 °C for 15 s (decreasing in temperature by 1 °C per cycle) and 72 °C for 30 s, followed by 40 cycles at 95 °C for 15 s, 55 °C for 15 s and 72 °C for 30 s.

**Table 1:** Primer sequences used in the analysis of cell-free fetal DNA

Primer name	Sequence (5′–3′)	Amplicon size (bp)
TDIF	CTGAGCGTCATCTGCCCC	127
TDIR	TGCGTCACTGTACACCTTGC	
TDIIF1	CGTGCACAACCTCGACTACTAC	120
TDIIR1	CTGGGAGGGTGTGGGAAG	
TDIIF2	ACGTGATGAAGATCGCAGACT	125
TDIIR2	AGGCGTCCTACTGGCATGA	

### Restriction enzyme analysis

For screening of samples for c.1948G>A mutation, 5 U of BbsI enzyme (New England Biolabs, Ipswich, MA, USA) was used to digest 25 μL of PCR product at 37 °C for 2 h. For detection of the c.742>T transition, 10 U of AfeI, BsiHKAI or DraIII enzymes was used to digest 25 μL of DNA at either 37 °C (AfeI and DraIII) or 65 °C (BsiHKAI) for 2 h. Digests were run on a 3% polyacrylamide gel and visualised with ethidium bromide. For the case where the mutation analysis was negative, the *RASSF1A* assay,^20^ as modified for use in clinical practice,^21^ or the *SRY* assay was used to confirm the presence of cffDNA.

## RESULTS

### Sonographic findings in TD

**Figure 2:**
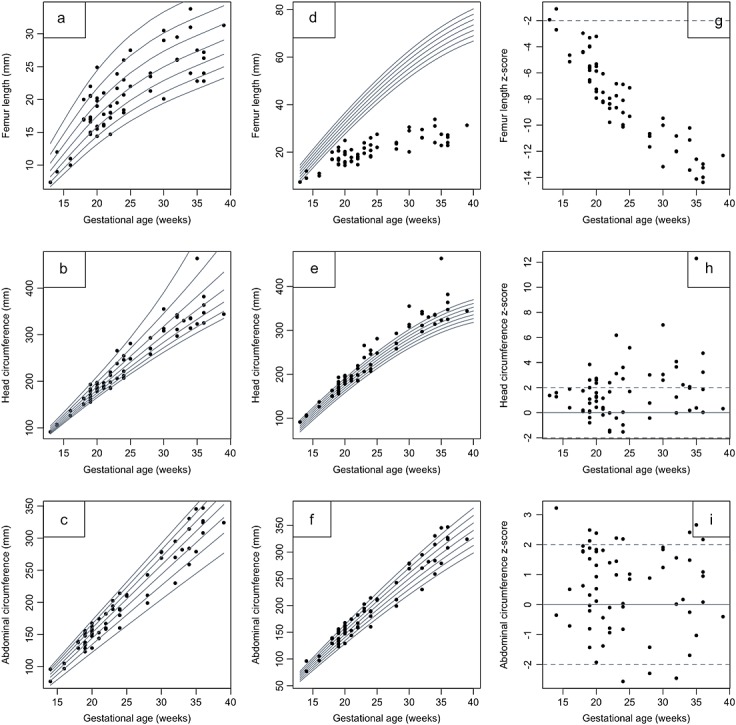
Fetal size charts for femur length (a), head circumference (b) and abdominal circumference (c) against gestational age in fetuses with thanatophoric dysplasia. Note the curvature in the femur length chart as growth declines significantly from 20 weeks gestation. Comparison with normal fetuses is demonstrated by overlaying measurements from affected fetuses on charts of fetuses of normal size (d–f, respectively) (derived from Chitty *et al*.^15^–^17^) and by plots of *Z*-scores showing the deviation from the normal range (g–i, respectively)

Forty-two cases with a confirmed diagnosis of TD had been scanned in our units. Presentation was between 13 and 32 weeks. In six cases, serial measurements were available. Charts were constructed using all available measurements treated cross-sectionally. Those for the head, AC and femur length are shown in Figure 2. Measurements of FL lay below the normal range from early in pregnancy (Figure 2d) with the difference between normal and TD femur length increasing with gestation (Figure 2g). The medians plotted against gestation were slightly curved for FL (Figure 2a) from around 20 weeks' gestation, after which time there was poor femur growth overall (Figure 2a and d). The fetal size charts for other long bones followed a similar pattern. The shapes of the HC (Figure 2b) and AC (Figure 2c) charts both followed the normal pattern (Figure 2e and f), although the HC tended to be greater in TD fetuses across gestation. *Z*-score plots (Figure 2g) comparing cases with the normal ranges^15^–^17^ show that the FL lies below the normal range for all except two cases. Both of these measurements were made at or before 14 weeks' gestation. By contrast, *Z*-plots confirm that the HC tends to be greater than normal across gestation (Figure 2h).

**Figure 3:**
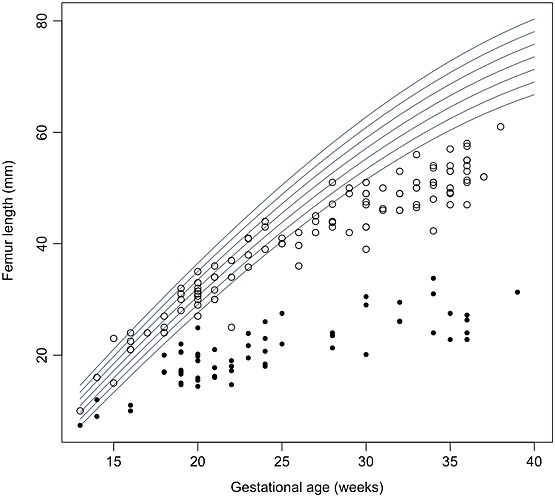
Comparison of femur length in fetuses with thanatophoric dysplasia (closed circle) or achondroplasia (open circle) with normal fetuses (derived from Chitty *et al*.^18^) showing the distinctive pattern for TD, with little increase in size from the mid second trimester and achondroplasia, which follows the normal femoral centiles until around 25 weeks' gestation when it then falls away from the normal centiles

Figure 3 shows the comparison of the femur length measurements in this cohort of fetuses with TD with a previously reported cohort of fetuses with achondroplasia.^18^ This demonstrates discrimination of femur length in these two conditions, with those of TD fetuses falling below those with achondroplasia at all gestations with the exception of one fetus with achondroplasia.

The reports describing additional ultrasound findings were available for all 42 of our cases (Table 2). A diagnosis of TD was suspected in 36 cases based on the sonographic features (Figure 1). In the other six cases, a diagnosis of a serious skeletal dysplasia was made, but no diagnosis was specified. In 13, the diagnosis was confirmed following invasive testing and molecular analysis that showed a mutation in the *FGFR3* gene and in a further three analysis of cffDNA extracted from maternal blood that confirmed the presence of the mutation. The diagnosis was confirmed in the remaining 26 cases by postnatal radiology or, in four of these, by molecular analysis that demonstrated the c.742C>T mutation in three cases and the c.1948A>G mutation in the fourth. Short limbs occurred in all cases with bowing of the femora, also a commonly reported feature, seen in 67% (28/42) of the cases. Frontal bossing was observed in 80% (24/30 cases where profile was visualised), cloverleaf skull in 31% (13/42), short fingers in 52% (16/31 where fingers were examined) and polyhydramnios in 31% (11/35 cases where amniotic fluid volume was recorded). Both small narrow chest and short ribs were described in 95% (35/37) of cases. These findings are compatible with descriptions available in the literature (Table 2), where short, bowed limbs in association with a small chest and relative macrocephaly are the most frequently reported findings. As with our series, there are also occasional reports of brain anomalies, including ventriculomegaly.

**Table 2:** Summary of the sonographic features reported in fetuses with thanatophoric dysplasia including the frequency of findings in the cases reported in this study

Sonographic finding	Our cases	Reports describing 1–3 cases	Chen *et al*.^22^	Tsai *et al*.^23^	Schramm *et al*.^5^	Overall frequency^a^	Overall frequency^a^ (%)
Total cases	42	40	4	9	40		
Gestational age (weeks)	13–32	11–30	18–31	20–34	16–35		
Increased nuchal translucency	2/3	6/7				8/10	80
Bowed femur	28/42	29/40	1/4	9/9		67/95	70
Short femur	42/42	40/40	4/4	9/9	40/40	137/137	100
Macrocephaly	23/39	15/17	1/4	6/9	20/38	65/107	61
Cloverleaf skull	13/42	11/40	1/4			24/86	30
Narrow thorax	35/37	38/38	4/4	9/9	37/40	123/128	96
Frontal bossing	24/30	6/8	4/4	9/9		43/51	84
Short fingers/trident hand	16/31					16/31	52
Increased AF	11/35	10/12		6/9		27/46	59
Other features:							
Ventriculomegaly	4	5					
ACC		1					
Talipes	2	3					
Redundant skinfolds		3					

AF, amniotic fluid; ACC, absence of the corpus callosum.

a Frequency only includes cases where there was evidence in the paper or, for our cases, the ultrasound report that a particular feature was examined.

### Development of cffDNA assays for TD

**Figure 4:**
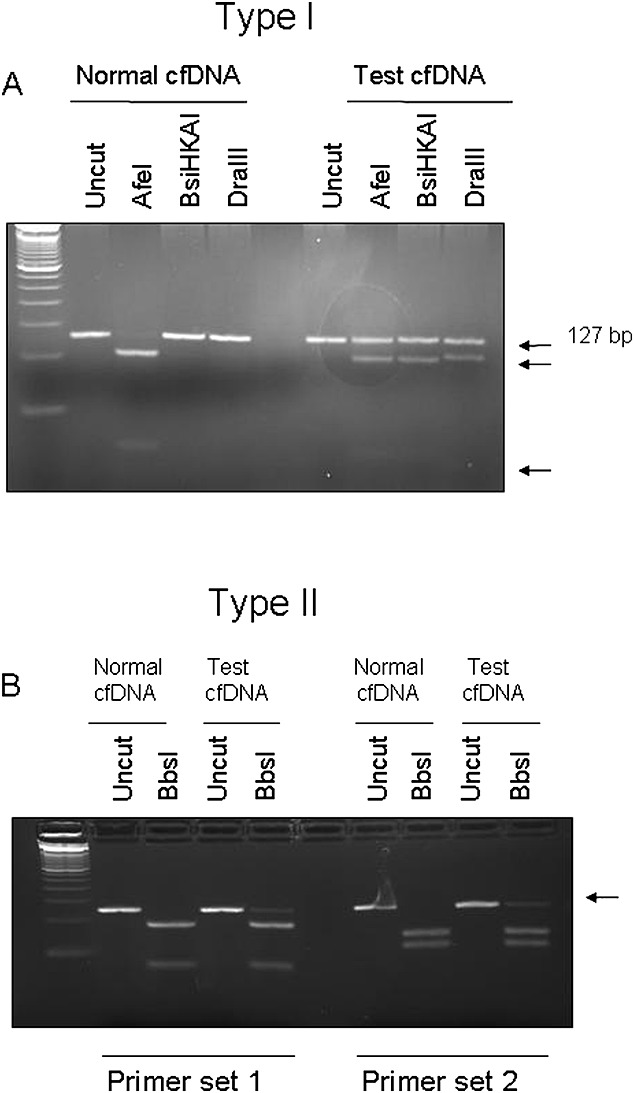
Restriction digest of PCR products for two thanatophoric dysplasia (TD) mutations. (A) The c.742C>T type I TD mutation was detected using PCR followed by digestion with AfeI, BsiHKAI and DraIII. cffDNA from a woman carrying an unaffected fetus is digested with AfeI (lane 3) but remains uncut using BsiHKAI (lane 4) and DraIII (lane 5); conversely, in an affected fetus, the AfeI site is destroyed, leaving some of the cffDNA undigested (lane 8), and a BbsI site (lane 9) and a DraIII site (lane 10) are created. (B) The c.1948A>G mutation was detected using digestion with the BbsI enzyme and two different primer sets. In the presence of an unaffected fetus, all cffDNA is digested by BbsI (lanes 3 and 8), whereas with an affected fetus, the BbsI restriction site is destroyed, leaving some of the cffDNA uncut (lanes 5 and 10)

There were three cases where maternal blood was taken for analysis of cffDNA because of suspicious ultrasound findings. In one of these families, blood was also taken in a subsequent pregnancy and cffDNA analysed (Table 3). The assay for TDI was optimised using gDNA from a chorionic villus sampling (CVS) sample from a fetus known to have a c.742C>T transition in the *FGFR3* gene, as well as gDNA from a normal control sample. The amplicon produced by primer set TDI is 127 bp in size. A normal DNA sample will be cut by AfeI (producing fragments of 26 and 101 bp) whilst remaining undigested by both BsiHKAi and DraIII (Figure 4a). In contrast, a sample that is heterozygous for the mutation will have one allele that remains uncut by AfeI but will be digested by BsiHKAi to yield fragments of 27 and 100 bp and by DraIII to yield fragments of 24 and 103 bp.

**Table 3:** Summary of cases where analysis of cffDNA was performed with details of the analysis

Indication	Mutation	GA	cffDNA	cffDNA presence confirmed	Comments
USS	c.742C>T (p.Arg248Cys)	21^+1^	+	n/d	Confirmed on amniocytes and PM tissues
USS	c.742C>T (p.Arg248Cys)	36^+4^	+	*RASSAF1A*	Confirmed on amniocytes
USS	c.1948A>G (p.Lys650Glu)	19	+	*RASSAF1A*	Confirmed on skin at PM
Past history	c.1948A>G (p.Lys650Glu)	12	−	*SRY*	Normal live birth

cffDNA, cell-free fetal DNA; GA, gestational age; USS, ultrasound; n/d, not done; PM, post-mortem.

PCR products from type I TDs or control plasma were examined for the presence or absence of the AfeI, BsiHKAI and DraIII restriction sites (Figure 4a). In all control plasmas, the DNA was digested by AfeI and not by BsiHKAI or DraIII, as expected. In the two type I TD plasma samples, the maternal contribution was for the same as that in the normal plasma, whereas one of the fetal alleles had lost the AfeI restriction site and gained a BsiHKAI and a DraIII site.

The A>T transition at nt 1948 eliminates a BbsI restriction site. Two sets of PCR primers were designed to amplify this region of DNA, the first producing an amplicon of 120 bp, the second set producing a 125 bp fragment. A homozygous unaffected sample will be digested by BbsI, producing fragments of 32 and 88 bp with primers TDII1 and 56 and 69 bp with primers TDII2 (Figure 4b). A heterozygous mutation will cause one of the fetal alleles to lose the restriction site, resulting in the presence of an additional uncut band. As before, this assay was optimised on gDNA (data not shown), then one normal and one TDII plasma sample were examined for the presence of the mutation. By using both sets of primers, the control plasma DNA was cut with BbsI, whereas in the affected sample, there was an extra band representing the uncut mutant allele.

## DISCUSSION

In this study, we have refined the range of sonographic features seen in TD and provided charts of fetal size, which clearly help distinguish this lethal condition from other conditions such as achondroplasia (Figure 3), one of the conditions most commonly confused with TD.^24^ We have demonstrated that limb shortening in TD is sonographically apparent from as early as 13 weeks' gestation, with minimal change in size after 20 weeks. Similarly, HC is increased throughout gestation, a feature that is present as early as the first trimester. We recognise that the comparisons are made with charts of fetuses of normal size that are derived from cross-sectional data, whereas the TD measurements are a mix of longitudinal and cross-sectional data. However, we feel that it is appropriate to compare with these charts as they are the ones in common use and would be used in clinical practice to assess measurements of an affected fetus.

Earlier series report that around 40% of osteochondrodysplasias are correctly diagnosed by ultrasound in the prenatal period.^6^–^10^,^25^,^26^ By using ultrasound scan alone, the diagnosis of TD was suspected in 86% of the cases in our cohort. This is consistent with other studies that investigated the accuracy of prenatal diagnosis of TD using ultrasound (88% in Schramm *et al*.,^5^ 70% in Sharony *et al*.,^6^ 50% in Tretter *et al*.,^7^ 40% in Gaffney *et al*.,^8^ 75% in Doray *et al*.^9^ and approximately 50% in Krakow *et al*.^10^).

Published reports suggest that it is possible to accurately predict that cases presenting with sonographic evidence of a skeletal dysplasia are likely to be lethal by evaluation of chest size and configuration.^10^,^27^ Others have used a femur length/AC ratio <0.16 as a sensitive threshold for predicting lethal skeletal dysplasia.^28^ In our series, we were able to evaluate this ratio in 35 cases – these and all TD cases reported in the literature that included the femur length/AC ratio had a FL/AC ratio of <0.16. Predicting lethality may be possible and reliable, but definitive confirmation of the underlying pathology is critical for accurate parental counselling with regard to recurrence risks as well as the management of future pregnancies. Traditionally, this can be carried out using molecular analysis of chorionic villi or amniocytes following invasive testing,^11^,^29^ or by postnatal radiology. The differential diagnosis for TD is broad and will vary with gestation (Table 4). In early pregnancy, the main differentials will include other dominantly inherited conditions such as spondylo-epiphyseal dysplasia congenita^30^ but will also include conditions such as the short-ribbed polydactyly syndromes that are inherited in an autosomal recessive fashion.^4^ With the increasing use of early ultrasound, detection of features suspicious of TD at a time when a surgical termination of pregnancy is an option will increase. This management pathway precludes the use of postnatal radiology for confirmation of diagnosis and restricts options to molecular analysis following invasive prenatal testing or analysis of fetal products after termination.

**Table 4:** Aids to the differential diagnosis of thanatophoric dysplasia (TD)

	TD	Achondroplasia	Jeunes asphyxiating thoracic dystrophy	SEDC	Achondrogenesis	OI Type II A/C	OI Type IIB	OI Type III	Hypophosphatasia	Majewski	Saldino-Noonan
Femur length (<20 weeks)	<<3rd	Normal	<3rd	<3rd	<<3rd	<<<3rd	5th	5th	<3rd	<3rd	<3rd
Femur length (>20 weeks)	<<<3rd	Normal until >25 weeks when growth falls	<3rd	<<3rd	<<<3rd	<<<3rd	<5th	<5th	<3rd	<3rd	<3rd
Head circumference	>95th	Relative macrocephaly	Normal	50th–95th	Relatively large	Normal	Normal	Normal	Normal	Normal	Normal
Abdominal circumference	Normal	In normal range	Normal	Normal	Relatively large	Normal	Normal	Normal	Normal	Normal	Normal
Frontal bossing	+	+	−	−	−	−	−	−	−	−	−
Hypomineralisation of vertebral bodies	−	−	−	1st trimester only	+	−	−	−	−	−	−
Short fingers/trident hand	+	+	+ Polydactyly	−	−	−	−	−	−	Polydactyly	Polydactyly
Small chest	+/−	+/−	+	+ (Short)	+	+	Mild	−	+	+	+
Ribs	Short	−	Short	Normal	Short	Beaded	Occasional beading	Normal		Short	Short
Polyhydramnios	+	+/−	−	−	+	+	−	−	+/−	−	−
Hypomineralisation of cranium	−	−	−	−	+	+	Mild	−	+	−	−
Limb fractures	−	−	−	−	−	+	+	+ Often confined to femora	+/−	−	−

SEDC, spondylo-epiphyseal dysplasia congenital.

In our experience, many women faced with the diagnosis of a lethal condition in their pregnancy are reluctant to undergo invasive diagnostic testing, and many will opt for a surgical approach to ending the pregnancy if possible. Thus, the availability of NIPD by analysis of cffDNA in maternal blood offers the potential for a very useful adjunct to prenatal diagnosis in this situation, allowing definitive diagnosis following a maternal blood sample. Other situations where NIPD may prove very useful include twin pregnancies where one twin is normal and the other has features of TD. Here, confirmation of the diagnosis is useful not just for genetic counselling but also to inform management without need for an invasive test that carries a risk to the normal fetus. Finally, whilst presentation in the third trimester is uncommon with widespread use of routine second trimester anomaly scanning, targeted NIPD screening for mutations in the FGFR3 gene could readily distinguish TD from achondroplasia. The recurrence risk for TD in future pregnancies is small and is based on a gonadal mosaicism risk,^31^ as this is a lethal dominant condition. However, parents will often request definitive exclusion of a recurrence in future pregnancies. Analysis of cffDNA in maternal plasma offers an accurate and early definitive, but safe, approach to excluding a recurrence. In this series, we include one case with a past history of TD, who underwent NIPD at 12 weeks' gestation and received early confirmation of the absence of the mutation.

The data we present here have demonstrated that NIPD for TD using PCR-RED analysis of cffDNA is possible. Because there are several mutations causing TD, but few are amenable to restriction digest, other approaches may be more appropriate. For example, digital PCR array using allele-specific real-time PCR assays can be used to discriminate between wild type and mutant alleles for up to 12 mutations per fetus, or alternatively, next generation sequencing may be a useful approach. These methods will require detailed evaluation but, whichever method is used, when no mutation is detected, it will be important to determine the presence^20^,^21^ and quantity of cffDNA^32^ to avoid reporting false negative results. This will be of particular relevance when screening for a recurrence in maternal blood earlier in pregnancy when the levels of cffDNA are lower, 33 thereby increasing the risk of assay failure.

In conclusion, the charts of fetal size for fetuses with TD presented here will allow for improved sonographic distinction from other skeletal dysplasias. Definitive diagnosis of TD is possible by analysis of cffDNA in maternal plasma. These data will allow for safer, accurate confirmation of TD and will inform parental counselling and pregnancy management.

**WHAT'S ALREADY KNOWN ABOUT THIS TOPIC?**

- Thanatophoric dysplasia is a lethal skeletal dysplasia amenable to prenatal diagnosis using fetal ultrasound with molecular confirmation following an invasive procedure.

**WHAT DOES THIS STUDY ADD?**

- This study provides data to help distinguish thanatophoric dysplasia from other skeletal dysplasias and demonstrates the potential for safe, definitive molecular confirmation using cell-free fetal DNA in maternal plasma.
